# From Dermographism in Bracelets to Dermatophytosis in Circles: The First Case of Tinea Imbricata Diagnosed in Morocco

**DOI:** 10.7759/cureus.110862

**Published:** 2026-06-14

**Authors:** Nouha Mansar, Nada Naciri, El Mustapha El Mezouari, Redouane Moutaj

**Affiliations:** 1 Parasitology-Mycology Laboratory, Avicenna Military Hospital, Marrakech, MAR; 2 Faculty of Medicine and Pharmacy, Cadi Ayyad University, Marrakech, MAR; 3 Department of Dermatology and Venereology, Mohammed VI University Hospital, Marrakech, MAR

**Keywords:** case report, imported mycosis, marrakech, morocco, mycological diagnosis, tinea imbricata, trichophyton concentricum, tropical dermatophytosis

## Abstract

Tinea imbricata is a chronic superficial dermatophytosis caused by the anthropophilic dermatophyte Trichophyton concentricum and is typically characterized by pruritic concentric annular scaly lesions. The disease is endemic to regions of Southeast Asia, the Pacific Islands, and Latin America and remains exceptionally rare in North Africa. We report the first documented case of tinea imbricata diagnosed in Morocco in a 38-year-old Pakistani man residing in Louisiana, USA, who developed characteristic skin lesions while staying in Morocco. The lesions appeared as intensely pruritic concentric plaques involving the inner thighs. Direct microscopic examination and fungal culture findings were highly suggestive of Trichophyton concentricum as the causative organism. Initial treatment with oral terbinafine combined with topical antifungal therapy resulted in only partial clinical improvement. Complete remission was achieved after switching to oral itraconazole associated with topical antifungal treatment. This case highlights the importance of considering imported tropical dermatophytoses even in non-endemic settings.

## Introduction

Tinea imbricata is a chronic superficial dermatophytosis caused by Trichophyton concentricum, an anthropophilic dermatophyte that primarily infects humans [1]. Clinically, it is characterized by concentric annular scaly lesions associated with intense pruritus, producing the typical “imbricated” appearance of the disease [2]. This condition is mainly endemic in tropical and subtropical regions, particularly in Southeast Asia, the Pacific Islands, and certain areas of Central and South America [3].

Although commonly encountered in endemic regions, tinea imbricata remains exceptionally rare in North Africa and other non-endemic countries. Increased international travel and population migration have contributed to the emergence of imported tropical mycoses outside their usual geographic distribution [4]. Several studies conducted in endemic areas, including Indonesia, the Solomon Islands, Malaysia, and Mexico, have highlighted the persistent circulation of T. concentricum and the epidemiological importance of this infection in tropical communities [5].

In non-endemic settings, diagnosis may be delayed because clinicians are often unfamiliar with the disease and because its clinical presentation can mimic other dermatological conditions such as annular psoriasis, eczema, or tinea corporis. We report what is, to the best of our knowledge and based on the available published literature, the first documented case of tinea imbricata diagnosed in Morocco in a patient originating from an endemic tropical region. This case highlights the importance of considering rare imported dermatophytoses in patients presenting with atypical annular lesions and emphasizes the role of mycological investigations in establishing the diagnosis.

## Case presentation

A 38-year-old Pakistani man residing in Louisiana, USA, presented with pruritic skin lesions that appeared during a trip to Morocco. His medical history was unremarkable. The patient had no history of diabetes mellitus, immunodeficiency, or systemic corticosteroid use. He reported having participated in a pilgrimage approximately one month before arriving in Morocco. In addition, he stated that a cousin had experienced similar skin lesions for nearly two years, with a relapsing course requiring intermittent treatment during flare-ups.

**Figure 1:**
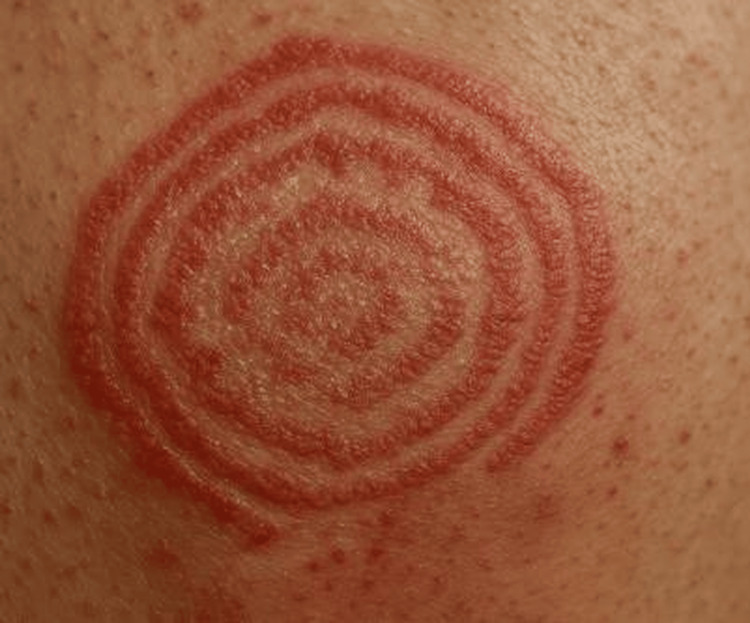
Typical concentric lesions of tinea imbricata

The lesions had been evolving progressively for almost three weeks before presentation. They initially developed on the inner aspects of both thighs and progressively evolved into concentric annular plaques with elevated, well-defined borders and central clearing (Figure 1).

The patient reported intense pruritus, particularly exacerbated by sweating. The history of similar lesions in a close family member may support intrafamilial transmission, which is consistent with the anthropophilic nature of the suspected pathogen.

**Figure 2:**
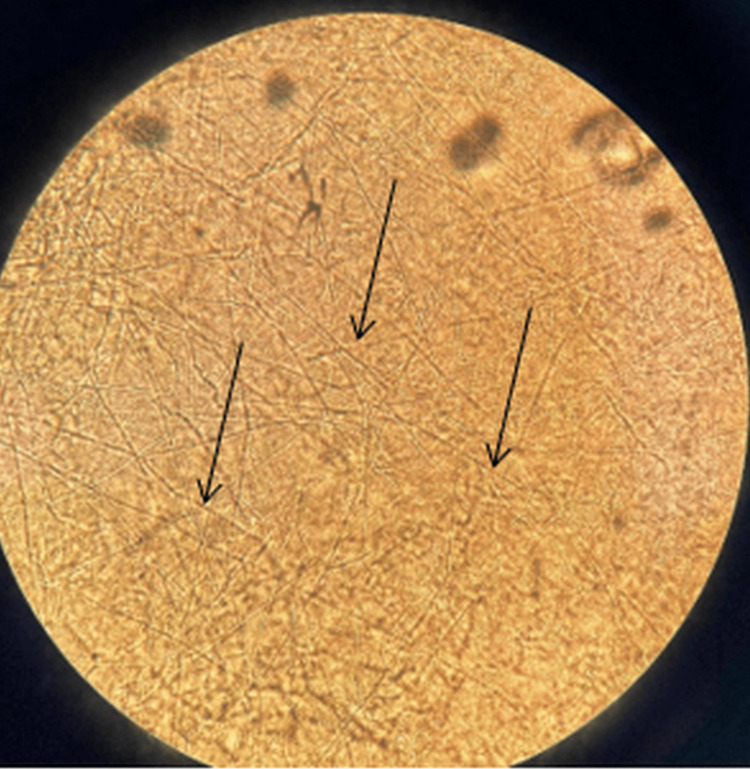
Direct microscopic examination of KOH-prepared skin scales showing septate fungal hyphae. Black arrows indicate septate fungal hyphae

Given this atypical clinical presentation, the patient was referred to the Parasitology-Mycology Laboratory of Avicenne Military Hospital in Marrakech for further mycological investigations. Skin scrapings were collected using a sterile scalpel blade from the active peripheral border of the lesions, where fungal proliferation is usually greatest. Direct microscopic examination after treatment with 30% potassium hydroxide (KOH) revealed hyaline, septate, branched fungal hyphae typical of dermatophytes, without arthroconidia (Figure 2).

**Figure 3:**
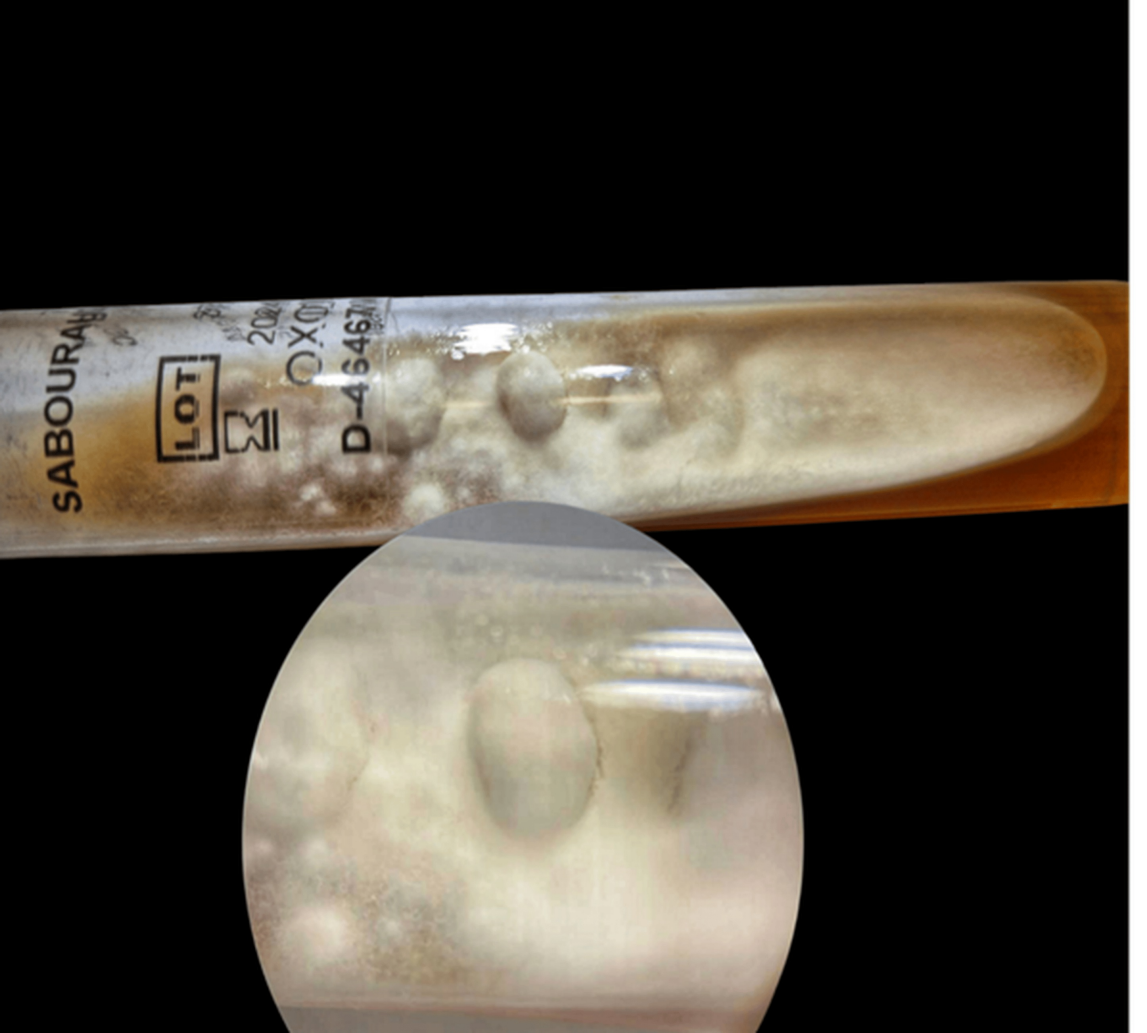
Macroscopic appearance of Trichophyton concentricum colonies on Sabouraud agar (obverse view)

Fungal culture was performed on Sabouraud agar supplemented with chloramphenicol and cycloheximide and incubated at 25°C. After eight days, irregular fluffy colonies with a cream-colored surface developed (Figure 3).

**Figure 4:**
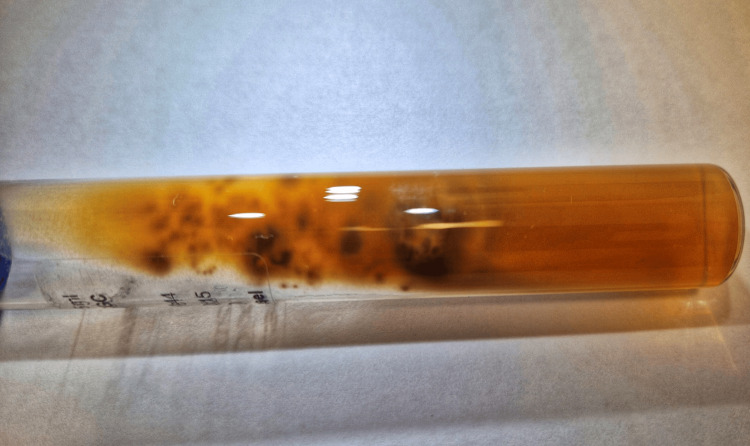
Macroscopic appearance of Trichophyton concentricum colonies on Sabouraud agar (reverse view)

Over time, the reverse side became yellow-brown to amber with marked central pigmentation (Figure 4).

**Figure 5:**
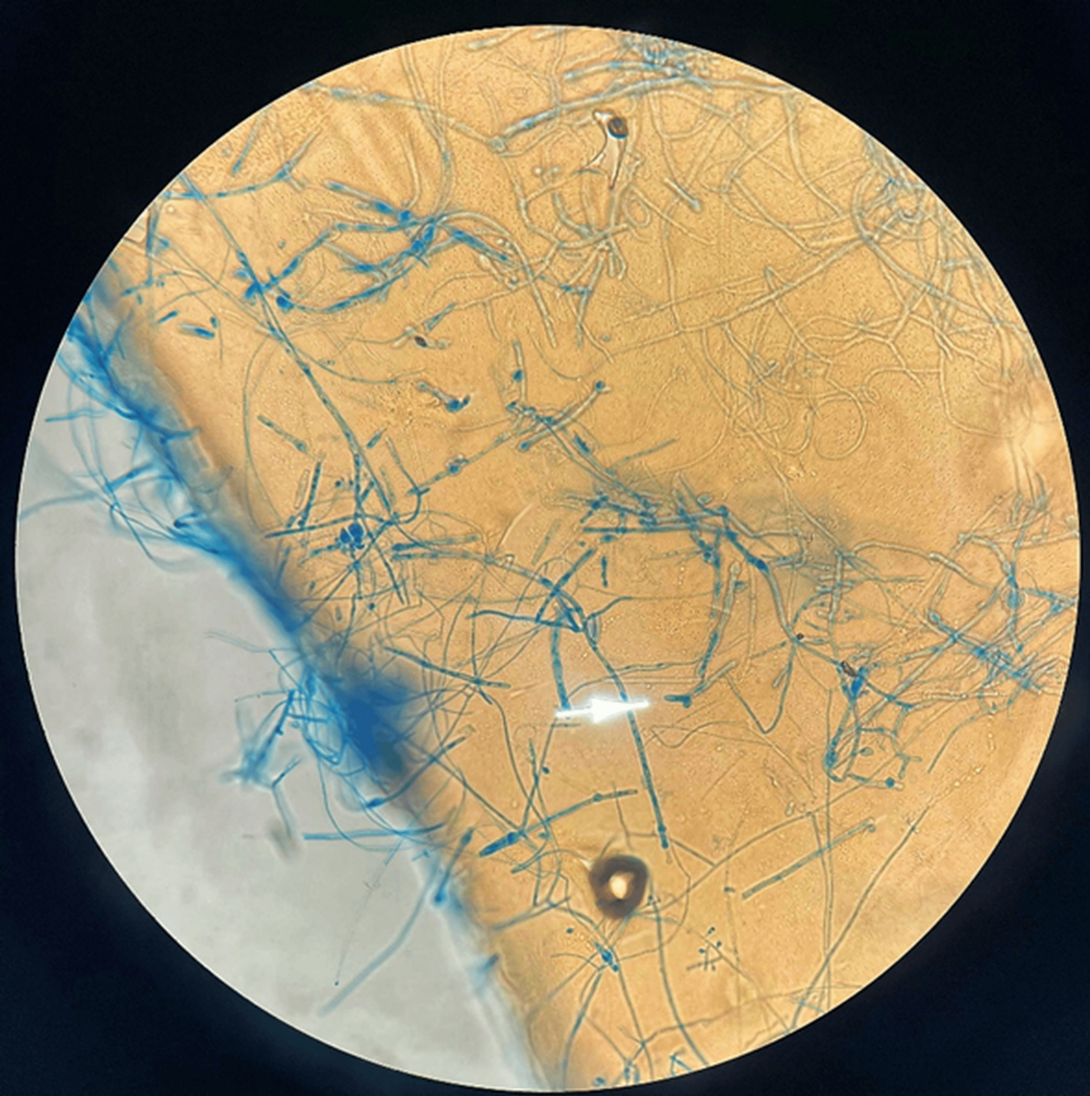
Microscopic examination with lactophenol cotton blue stain showing favic nails

**Figure 6:**
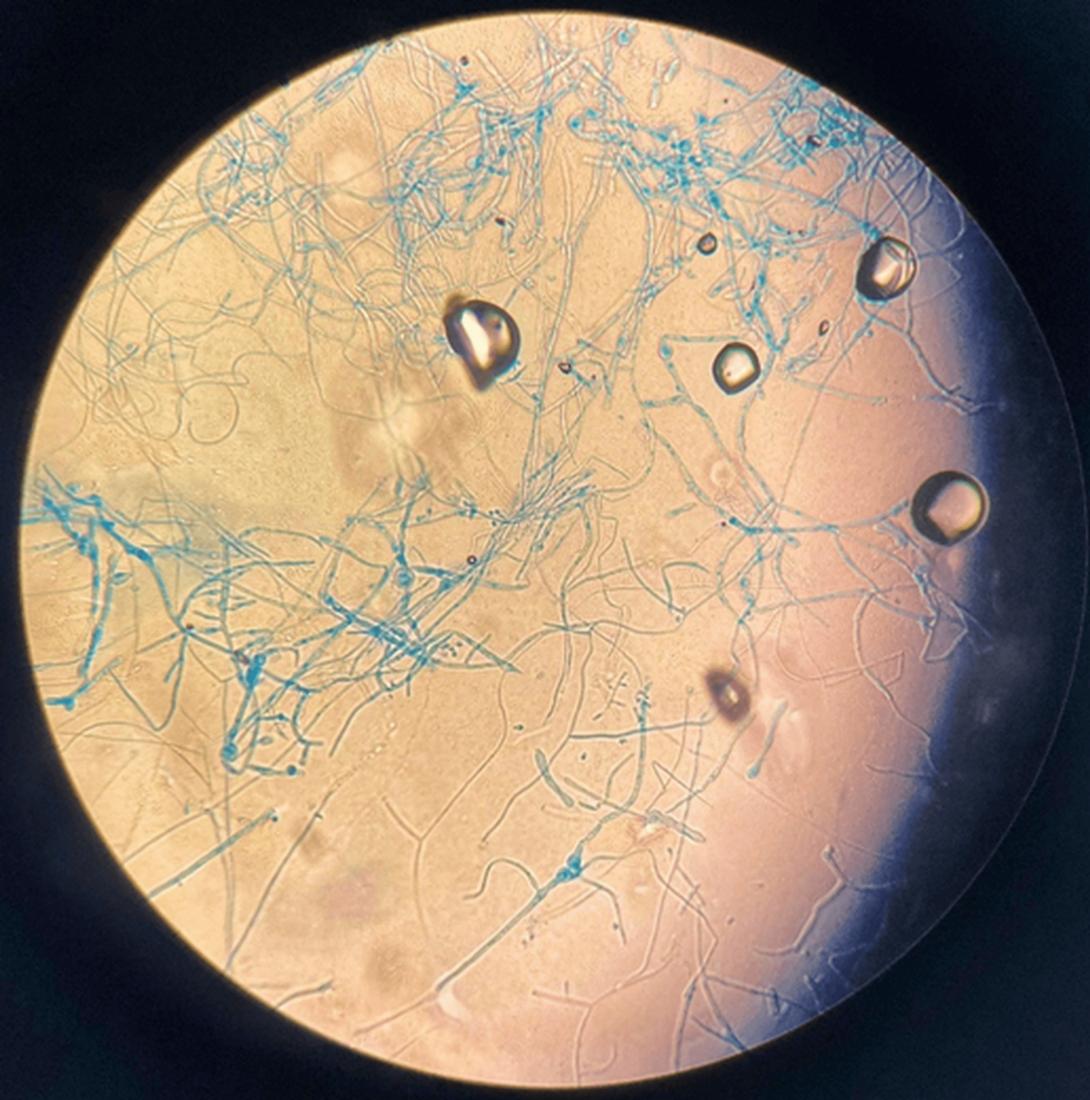
Microscopic examination showing “deer antler” hyphal configuration suggestive of Trichophyton concentricum

Microscopic examination using the flag technique in lactophenol cotton blue demonstrated large septate hyphae that were highly branched, tortuous, and irregular. Some hyphal tips exhibited a characteristic “deer antler” appearance suggestive of Trichophyton concentricum (Figures 5, 6).

**Figure 7:**
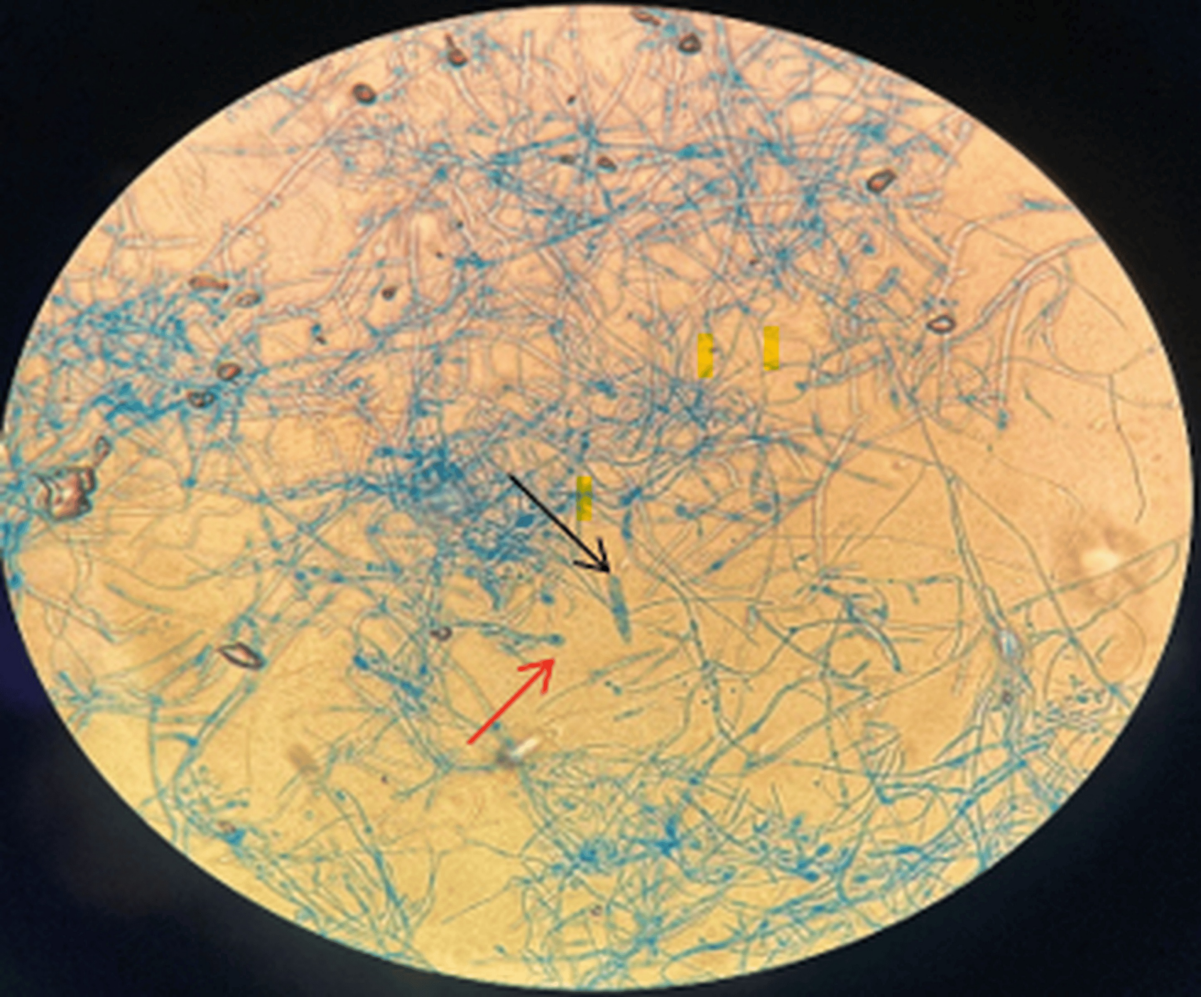
Microscopic examination showing thick septate hyphae associated with chlamydospores. The red arrow indicates chlamydospores, while the black arrow indicates macroconidia

Chlamydospores were frequently observed in older cultures, along with characteristic favic structures described as “favic nails” and “favic chandeliers” (Figure 7).

Although microconidia and macroconidia production was scarce, the overall clinical and morphological findings were highly suggestive of Trichophyton concentricum.

The patient was initially treated with oral terbinafine (250 mg/day) combined with topical terbinafine for four weeks. Despite partial clinical improvement, persistent active lesions remained, particularly in the inguinal folds. Due to this incomplete response, treatment was switched to oral itraconazole (200 mg/day for 14 days) while maintaining topical antifungal therapy. This therapeutic adjustment resulted in rapid and complete clinical remission, with no recurrence observed after four weeks of follow-up.

## Discussion

The present case highlights a rare form of dermatophytosis known as tinea imbricata, caused by Trichophyton concentricum, a strictly anthropophilic dermatophyte. Historically, this condition has mainly been reported in endemic regions such as Papua New Guinea, the Solomon Islands, the Philippines, Southeast Asia, the South Pacific, and Latin America [6]. However, sporadic cases are increasingly being identified in non-endemic regions due to globalization, migration, and international travel. To our knowledge and based on the available published literature, this may represent the first documented case of tinea imbricata diagnosed in Morocco.

Clinically, tinea imbricata is characterized by slowly progressive concentric annular lesions involving the trunk and limbs, often associated with pruritus that worsens with sweating [7]. In the present case, the concentric morphology of the lesions involving the thighs was highly suggestive. The history of similar lesions in a family member raises the possibility of shared environmental exposure or genetic susceptibility. Recent studies have demonstrated an association between CARD9 mutations and increased susceptibility to chronic dermatophyte infections [8]. However, no immunological or genetic investigations were performed in our patient; therefore, the potential contribution of such factors remains speculative. Such immunological susceptibility, combined with an impaired local immune response, may contribute to the chronic evolution of the disease and occasional therapeutic difficulties reported in some patients [9].

In endemic regions, the clinical presentation alone may strongly suggest the diagnosis. However, in non-endemic areas, tinea imbricata may mimic several inflammatory dermatoses including annular psoriasis, eczema, erythema gyratum repens, or dermographism [10]. The differential diagnosis also includes tinea corporis, pityriasis rosea, erythema annulare centrifugum, and other figurate erythemas. In the present case, the characteristic concentric scaling pattern, intense pruritus, epidemiological background, and supportive mycological findings strongly favored the diagnosis of tinea imbricata. Consequently, mycological investigations remain essential. In our case, direct microscopic examination after potassium hydroxide clarification demonstrated hyaline, septate, branched fungal hyphae without arthroconidia, findings consistent with dermatophyte infection [11]. Although frequently underestimated, direct examination represents a rapid and valuable diagnostic tool for the identification of unusual dermatophytes.

Culture on Sabouraud agar supplemented with chloramphenicol and cycloheximide revealed velvety cream-colored colonies with amber pigmentation on the reverse side after eight days of incubation, findings highly suggestive of Trichophyton concentricum [12]. Microscopic examination using lactophenol cotton blue staining demonstrated highly branched tortuous septate hyphae with characteristic “deer antler” configurations, along with favic structures described as “favic nails” and “favic chandeliers” [13]. Chlamydospores were also observed in older cultures. Altogether, these clinical and morphological features were highly suggestive of Trichophyton concentricum.

In the present case, the diagnosis relied on the characteristic clinical presentation, direct microscopic examination, and fungal culture findings. Although molecular methods were not available, techniques such as PCR targeting the internal transcribed spacer region, sequencing of marker genes including TUB2 or squalene epoxidase, multiplex real-time PCR, and matrix-assisted laser desorption/ionization-time of flight mass spectrometry have significantly improved dermatophyte identification, particularly in atypical or resistant cases [14].

Treatment of tinea imbricata generally requires prolonged systemic antifungal therapy. Historically, griseofulvin has been considered the reference treatment [15]. Currently, terbinafine is widely recommended because of its favorable pharmacokinetic profile and good cutaneous diffusion. Nevertheless, emerging terbinafine resistance associated with mutations affecting squalene epoxidase has increasingly been reported, particularly in Southeast Asia and Oceania [16]. Itraconazole therefore represents an effective alternative in cases of confirmed resistance or therapeutic failure [17].

In our patient, initial treatment with oral and topical terbinafine resulted only in partial improvement. However, antifungal susceptibility testing and molecular analysis of resistance-associated mutations were not performed; therefore, terbinafine resistance could not be confirmed. Alternative explanations include disease chronicity, host-related factors, variability in drug exposure, or other factors influencing therapeutic response. Switching to oral itraconazole led to complete clinical remission without recurrence during follow-up. This therapeutic response emphasizes the importance of close clinical monitoring and early therapeutic adaptation in cases of incomplete response to first-line antifungal therapy.

## Conclusions

To the best of our knowledge and based on the available published literature, this may represent the first documented case of tinea imbricata diagnosed in Morocco. This case highlights the growing importance of mycological surveillance in the context of increasing globalization and population mobility. The emergence of a tropical dermatophytosis in a non-endemic Mediterranean country emphasizes not only the essential role of specialized Parasitology-Mycology laboratories in identifying uncommon fungal pathogens, but also the importance of adapting therapeutic strategies according to clinical response.

The diagnosis in this case was based on characteristic clinical findings, direct microscopic examination, and fungal culture findings highly suggestive of Trichophyton concentricum, although molecular confirmation was not available. This case underlines the need for early diagnosis, personalized antifungal management, and increased awareness among healthcare professionals regarding imported and exotic fungal infections, whose incidence may continue to rise in the future.
